# Impact of the COVID-19 pandemic on paediatric orthopaedic trauma workload in central London: a multi-centre longitudinal observational study over the “golden weeks”: The COVid Emergency Related Trauma and orthopaedics (COVERT) Collaborative

**DOI:** 10.1080/17453674.2020.1807092

**Published:** 2020-08-24

**Authors:** Kapil Sugand, Chang Park, Catrin Morgan, Rory Dyke, Arash Aframian, Alison Hulme, Stuart Evans, Khaled M Sarraf, Camilla Baker, Katharine Bennett-Brown, Henry Simon, Edward Bray, Lily Li, Noel Lee, Nadia Pakroo, Kashed Rahman, Andrew Harrison

**Affiliations:** a Imperial College Healthcare NHS Trust, London; b Chelsea and Westminster Hospital, London, UK

## Abstract

Background and purpose — The COVID-19 pandemic has been recognised as an unprecedented global health crisis. This study assesses the impact on a large acute paediatric hospital service in London, evaluating the trends in the acute paediatric orthopaedic trauma referral caseload and operative casemix before (2019) and during (2020) COVID-19 lockdown.

Patients and methods — A longitudinal retrospective observational prevalence study of both acute paediatric orthopaedic trauma referrals and operative caseload was performed for the first 6 “golden weeks” of lockdown. These data were compared with the same period in 2019. Statistical analyses included median (± median absolute deviation), risk and odds ratios as well as Fisher’s exact test to calculate the statistical significance, set at p ≤ 0.05.

Results — Acute paediatric trauma referrals in 2020 were reduced by two-thirds compared with 2019 (n = 302 vs. 97) with a halving risk (RR 0.55) and odds ratios (OR 0.43) of sporting-related mechanism of injuries (p = 0.002). There was a greater use of outpatient telemedicine in the COVID-19 period with more Virtual Fracture Clinic use (OR 97, RR 84, p < 0.001), and fewer patients being seen for consultation and followed up face to face (OR 0.55, RR 0.05, p < 0.001).

Interpretation — The impact of the COVID-19 pandemic has led to a decline in the number of acute paediatric trauma referrals, admissions, and operations during the COVID period. There has also been a significant change in the patient pathway with more being reviewed via the means of telemedicine to reduce the risk of COVID-19 transmission and exposure. More work is required to observe for similar trends nationwide and globally as the pandemic has permanently affected the entire healthcare infrastructure.

The novel coronavirus SARS-COV-2 (COVID-19) will be documented as one of the most unprecedented pandemics within modern history. It was first reported in December 2019 with the first patient hospitalised in the city of Wuhan, China (Wu et al. 2020). Declared a pandemic and a global public health emergency by the World Health Organization (2020), as of July 25, 2020 there are over 15 million reported cases and over half a million mortalities.

## British response to the pandemic

The English government responded by implementing stringent social distancing and lockdown measures by mid-March. As of March 23, 2020, all members of the public were required to stay at home unless for essential purposes (UK Government 2020a, b). In response to the National Health Service (NHS) emergency declaration (NHS England 2020a, b), a collaborative effort between NHS England, NHS Improvement, the Royal Colleges, the British Orthopaedic Association (BOA), and the British Society for Children’s Orthopaedic Surgery (BSCOS) resulted in a compilation of guidelines to standardise and protocolise care on a national level during the COVID-19 outbreak (British Orthopaedic Association 2020).  

## Burden of paediatric trauma

Trauma is a major cause of mortality and morbidity within the paediatric population in the face of a concurrent decline in paediatric trauma services that has been recognised globally (Danseco et al. 2000, Spady et al. 2004, Peden et al 2008, Tuason et al. 2009). Recent studies of the UK population have found fracture rates in children to be between 76 and 137 per 10,000 person years (Orton et al. 2014, Moon et al. 2016). Although there is complete suspension of elective adult care, semi-elective and urgent elective paediatric care are still deemed to be essential during the pandemic. The unique consequence of the COVID-19 pandemic has meant that schools and nurseries have had to stay closed as a result of the lockdown from March and children, like adults, have had to adjust to life in the constraints of their own homes.

## Objective

This study aimed to evaluate the impact of the COVID-19 pandemic in one of the largest central London multi-centre paediatric hospital services, evaluating the trends in acute orthopaedic trauma referral caseload and operative casemix before (2019) and during the COVID-19 lockdown (2020) from mid-March to the end of April (i.e., over a period of 6 weeks).

## Alternative hypothesis

The alternative hypothesis was that there will be a difference in the prevalence of acute orthopaedic referrals, orthopaedic trauma casemix, and follow-up due to social distancing/lockdown. As an unintended consequence, not being able to leave the household due to social distancing and the lockdown, it was hypothesised that children may be less at risk of accidents and injuries during the COVID-19 pandemic.

## Patients and methods

### Patient sampling

All acute referrals, operative notes, inpatient medical records, and discharge summaries were accessed using the electronic medical system within the hospitals as well as from the eTrauma online system (Open Medical Ltd, London, UK), allowing for consistency in data collection. This real-time online database is accessed by those in the trauma and orthopaedic department and records all acute referrals and operations, both retrospectively and prospectively.

### Hospital sites

St Mary’s Hospital is a major trauma centre and equivalent to a level 1 trauma centre, managing the entire spectrum of orthopaedic and non-orthopaedic polytrauma. Chelsea and Westminster Hospital is a large district general hospital equivalent to a level 2 trauma unit, with a dedicated orthopaedic and plastics unit. Both sites have dedicated paediatric orthopaedic departments led by consultant/attending surgeons. Together, they both represent specialist tertiary centres for paediatric orthopaedic trauma and elective surgery.

### Study period

The study period was from the start of the government-imposed social distancing on the morning of March 17, 2020 (which includes the more stringent “lockdown” from the morning of March 24) to the April 28, 2020. This was compared with the same 6-week interval in 2019 when there were no such restrictions on social interaction.

### Inclusion criteria (for both years)

All acute orthopaedic trauma cases presenting to the Emergency Department that were subsequently referred to the trauma orthopaedic department either to fracture clinic services or to the acute referral team were included, as were all orthopaedic trauma cases that required an operation within the defined study period 1 year apart. Those patients listed for an operation prior to the period of data collection were included in the final analysis. The study adhered to the STROBE guidelines.

### Exclusion criteria (for both years)

We excluded any patient aged over 18 years old. For operative trauma cases, those undergoing spinal procedures were excluded as the service is delivered jointly by the neurosurgery team. Any non-urgent semi-elective procedures were excluded from further analysis as this would inaccurately assess the impact of any social distancing measures on trauma workloads. Routine elective orthopaedic cases were excluded because this practice was suspended. 

### Data points

Demographics including age, sex, and ASA grades were recorded for all patients. Injury characteristics were recorded, including the anatomical location and whether the injury was open or closed. The mechanism of injury was categorised and whether or not the patient was referred as a trauma call. The nature of the operative procedure and the anaesthetic technique were recorded. Patients undergoing multiple procedures were recorded for every episode when they were taken to theatre.

### Statistics

All data were anonymised as well as being verified by 2 authors for accuracy. A Shapiro–Wilk test was conducted for normality, which indicated that the data ought to be treated non-parametrically. The median (± median absolute deviation) was calculated for both age and ASA grade. A Mann–Whitney U-test calculated for significance for continuous data. Both risk (or prevalence) and odds ratios were calculated for discreet datasets as well as a Fisher’s exact test for statistical significance, defined as p ≤ 0.05.

### Ethics, funding, and potential conflicts of interest

There were no ethical objections. Both centres gave permission for the use of the data. This study was assessed using the UKRI/MRC/NHS Health Research Authority Ethics Decision Tool and was considered an ‘audit/not research’; and therefore it was not subject to further ethical review by the NHS Research Ethics Committee (NHS REC).

All patient details remained anonymised. All information was stored on NHS encrypted servers. This study received no funding. The authors have no conflict of interests to declare.

## Results

A comparison between the two cohorts has been tabulated (Table 1).

**Table 1.** Results from the acute referrals and requirement for surgery between both time intervals pre-COVID 2019 and COVID 2020. Values are n (%) unless otherwise specified

	Pre-COVID	COVID	
	N = 302	N = 97	p-value
Demographic			
Male	183 (61)	53 (55)	0.3
Female	119 (39)	44 (45)	
Median age (MAD)	10 (3)	7 (4)	0.02
Median ASA (MAD)	1 (0)	1 (0)	0.8
Injury			
Upper limb	201 (67)	67 (69)	
Lower limb	89 (29)	23 (24)	
Pelvis	3 (1)	1 (1)	
Infection	6 (2)	6 (6)	
Other	3 (1)	0 (0)	
Mechanism of injury			
Sporting	114 (38)	20 (21)	0.002
Fall	141 (47)	50 (52)	
Fall from height >1.5m	8 (3)	6 (6)	
Road traffic accidents	4 (1)	2 (2)	
Pathological	0 (0)	2 (2)	
Other	21 (7)	8 (8)	
Laceration	0 (0)	1 (1)	
Infection	6 (2)	4 (4)	
Trampoline	5 (2)	3 (3)	
N/A	0 (0)	1 (1)	
Open injury	4 (1)	4 (4)	
Trauma call	3 (1)	1 (1)	
Admission	33 (11)	18 (19)	
Operative management	48 (16)	16 (16)	
Safeguarding	25 (8)	11 (11)	
Comorbidity			
ADHD	0 (0)	1 (1)	
Asthma	7 (2)	3 (3)	
Osteogenesis imperfecta	1 (0)	1 (1)	
None	276 (91)	90 (93)	
Autism	2 (1)	1 (1)	
Eczema	1 (0)	1 (1)	
Leukaemia	1 (0)	0 (0)	
Epilepsy	2 (1)	0 (0)	
G6PD deficiency	1 (0)	0 (0)	
Sickle cell	1 (0)	0 (0)	
Type 1 diabetes mellitus	3 (1)	0 (0)	
Tuberculosis	1 (0)	0 (0)	
WPW syndrome	1 (0)	0 (0)	
Febrile convulsions	2 (1)	0 (0)	
Migraine	1 (0)	0 (0)	
Thalassaemia	1 (0)	0 (0)	
COVID status			
Negative		1 (1)	
Positive		2 (2)	
Not tested		94 (97)	
Clinic outcome			
Discharge	164 (54)	59 (61)	
Referral	2 (1)	2 (2)	
N/A	103 (34)	9 (9)	
Follow-up	23 (8)	22 (23)	0.001
DNA	10 (3)	5 (5)	
Clinic pathway			
Hybrid – face to face			
+ virtual clinics	0 (0)	4 (4)	
Discharged from face			
to face clinic	2 (1)	2 (2)	
Discharged via virtual clinic	0 (0)	26 (27)	< 0.001
Face to face clinic	290 (96)	47 (48)	< 0.001
N/A	9 (3)	10 (10)	
Referral to virtual clinic	0 (0)	9 (9)	< 0.001
Referral to hand therapy	1 (0)	0 (0)	
Number of follow-ups to date	2	1	< 0.001
Days to discharge	0	0	0.7
Days to first orthopaedic review	7	0	< 0.001

### Prevalence, risk, and odds ratios

Table 2 outlines the prevalence and odds ratios alongside their 95% confidence intervals and statistical significance. The risk ratio is synonymous with the prevalence ratio. Only the statistically significant ratios were included. There was a significant reduction in the odds of sporting-related mechanism of injuries (by 57%) in 2020 compared with 2019. Although the prevalence and odds ratios had significantly increased for follow-up during the COVID era, the odds of face-to-face consultations had a significant drop of 95%. The follow-up appointments could now be conducted safely using telecommunications such as a virtual fracture clinic, which increased prevalence 59-fold. Consequently, there was also a strong trend to discharge patients safely from the virtual fracture clinic with an odds ratio of 224.

### Comment on alternative hypothesis

The alternative hypothesis was not rejected. During the COVID period there was a reduced number of acute orthopaedic referrals consisting of younger patients (p = 0.02) presenting with half odds of sporting-related injuries (p = 0.002). Patients were at greater odds of being followed-up (p < 0.001), while face-to-face consultations halved in favour of the virtual fracture clinic with the capability of safe discharge (p < 0.001).

## Discussion

### Shift in referrals

The data supported the alternative hypothesis, which demonstrated that in the “golden weeks” following the governmental order to socially distance and isolate, there has been a notable reduction in paediatric injuries compared with last year. There were only 97 referrals following the introduction of social distancing measures in 2020 compared with 302 in 2019. This represents a 68% reduction in paediatric injuries. This reduction is likely a direct consequence of the social distancing measures implemented on a national scale. As the aetiology of paediatric fractures is linked to physical activity, often outdoors, the lockdown has significantly reduced the incidence of paediatric injuries (Schalamon et al. 2011, Joeris et al. 2014).

There may also be a shift in patient behaviour, with parents being more anxious about attending hospital due to the risk to themselves and their child of contracting COVID-19. Those who would have previously promptly sought acute services may have delayed and attempted to treat injuries at home. It is possible a number of these injuries or symptoms may have self-resolved without the need for urgent medical attention. This may represent an explanation for the reduction in the number of acute orthopaedic presentations to the Emergency Department seen in 2020.

### Demographic and mechanism

The general demographic of those presenting with injuries changed between the 2 periods, with a significantly younger median age (p = 0.02) in 2020 and more girls (p = 0.3). The younger cohort during the pandemic may reflect a less risk-averse population sample leading to trauma, or more concerned parents with a lower threshold of presenting to ED for reassurance, especially with a lower ability of the child to communicate his/her symptoms. There was no statistically significant difference in the comorbidities in those being referred, with the same median ASA grade of 1. The pattern of injury also remained generally unchanged with upper limb injuries being the most common at 67% and 69% respectively in 2019 and 2020. There was a significant decline in sporting-related injuries by 17% in 2020 (p = 0.002, RR = 0.55, OR = 0.43). This is unsurprising as following government guidelines all sports were banned during the lockdown and, even with the relative easing in mid-May, team sports remained forbidden. Falls remained the most common mechanism of injury in 2020, and these injuries can have occurred at home as well as during the single episode of exercise that the government allowed during lockdown.

### Non-accidental injuries (NAI)

NAI should always be considered in the paediatric population and the principles have not changed during the pandemic; healthcare workers must remain vigilant during times of increased stress and social isolation (NHS England 2020c). Indeed, with families under lockdown there have been reports of increased rates of domestic violence in households during the pandemic and this raises the concern for more NAI during the COVID-19 period (Alradhawi et al. 2020). In 2020 there were 3% more safeguarding referrals. Whilst there was no statically significant difference between the two periods, with school systems closed the usual pathways for the detection of the unusual and NAI are no longer in place. Nevertheless, there has not been a large increase in delayed presentation of substantial injuries that would prompt such a concern due to the effects of the pandemic. We are aware that delayed presentation is always a possibility that may have not been captured in the set time period for this study.

### Operative intervention

In 2020 there was an approximate 50% reduction in inpatient admissions and two-thirds reduction in those undergoing operative intervention. However, due to the overall reduction in referrals, the percentage of those admitted or undergoing operative intervention remained relatively unchanged. Against the backdrop of such a significant reduction in new acute trauma referrals, there is still a small percentage of substantial injuries that require admission and operative intervention in spite of social distancing and lockdown measures.

The BOA guidance published at the beginning of the pandemic recommended increased emphasis on the non-operative management of paediatric injuries during the COVID-19 pandemic (British Orthopaedic Association 2020). With the paediatric remodelling potential and remedial options in the form of later corrective surgery (e.g., for malunion), the guidelines recommended changes that deviated from standard practice for managing paediatric injuries. Although the new risks of COVID-19 were considered for the management of all patients, ultimately the decision to operate was based on the best interests of the child. In our practice no patient requiring operative intervention was denied this on the basis of COVID-19 alone for an acute issue. This was also enabled due to the overall reduction in referral numbers and operative cases, suggesting that a scenario did not occur whereby limited operative capacity was overwhelmed such that surgical treatment was deferred.

### Infection

Children represent approximately 2% of all COVID-19 cases but to reduce the risk of increased spread schools and nurseries closed during this period (Docherty et al. 2020). Subsequently, there is an expectation of a reduction in acute referrals for infection-related pathology in the paediatric population. The current literature suggests that children appear to be less susceptible to the effects of COVID-19, often displaying milder symptoms than their adult counterparts (Dong et al. 2020, Henry et al. 2020, Lu and Shi 2020, Ludvigsson 2020). Nevertheless, there have been a number of documented cases of a more serious associated illness similar to that of Kawasaki vasculitis (Harahsheh et al. 2020, Viner and Whittaker. 2020). The concern in the paediatric population is that COVID-19 may cause an increased incidence in the presentation of transient synovitis, or indeed COVID-related septic arthritis and osteomyelitis. In our study there has been an increase in overall referrals for infection, from 2% in 2019 to 6% in 2020. As the pandemic evolves and more information surfaces, further investigation is required to observe the influence of COVID-19 on paediatric musculoskeletal infections. Although we have found an increase in those being referred for infection, this was statistically insignificant. However, a larger and broader population sample may suggest otherwise.

There were 2 patients who tested positive for COVID-19 via nasopharyngeal PCR swabs. Both patients were admitted and treated surgically for septic arthritis requiring operative washout of the hip and knee. The joint aspirate in the washout of the knee subsequently grew *Neisseria meningitis* and *Staphylococcus aureus* from the hip but no COVID-19 PCR was identified from the operative tissue sample. To our knowledge there has not been any literature investigating the link between COVID-19 and septic arthritis in children, but the fact that 2 COVID-positive paediatric patients in 2020 had washout of a major joint for septic arthritis certainly warrants further investigation.

### Patient pathway

The COVID-19 pandemic has meant that the pathway for managing paediatric patients has changed to minimise risk to both patient and family (British Orthopaedic Association 2020). The threshold for paediatric follow-up tends to be lower due to children being a vulnerable cohort without self-advocacy and with limited communication. Monitoring paediatric patients is an acceptable reason for continuing follow-up. The COVID-19 pandemic challenged this policy as most children infected with COVID-19 seem to develop a sub-clinical condition or mount a milder immune response and illness, with the exception of a few. Hence, they are more likely to act as vectors to contribute to viral transmission (Dong et al. 2020, Henry et al. 2020, Lu and Shi 2020, Ludvigsson 2020).

Against the backdrop of the pandemic, there was a significant increase in the prevalence and odds of follow-up despite these concerns. However, the justification for this increase was the introduction of virtual fracture clinic (VFC). The VFC can be conducted by surgeons remotely, whereby they review the clinical referral from the Emergency Department while assessing the electronic imaging. The decision tree consists of either follow-up or discharge. Patients can be seen in a face-to-face clinic to either review their progress, arrange for further imaging, or commence treatment. The other option is to discharge the patient either to physiotherapy/hand therapy/orthotics with patient information leaflets that can be posted or emailed to them.

It is due to its availability, accessibility, and affordability that the utilisation of telemedicine has been a key adaptation in the care of patients during the pandemic (Loeb et al. 2020, Tanaka et al. 2020). VFC can also help to avoid the “did not attend” (DNA) situation that was seen in 2019. Consequently, there was a substantial rise in the prevalence and odds of referring and discharging paediatric patients safely from VFC follow-up. Conversely, face-to-face consultation, as expected, was also almost halved with regard to prevalence but the odds of seeing the paediatric patient in the follow-up clinic were reduced by 95%. This was not only statistically significant but also clinically significant as reducing face-to-face consultations was vital in curbing viral transmission, especially in a vulnerable cohort. This was also combined with the number of follow-ups halving (p < 0.001).

Furthermore, more urgent reviews were also implemented with a swifter first orthopaedic review of 1-week to same-day review (p < 0.001). This is in keeping with the recommendations made by the BOA (2020). Due to the restructuring of services, same-day physiotherapy and hand therapy were offered to patients either face to face or via video consultations.

There is a concern regarding increased NAI during the lockdown, which may go unnoticed, especially with increasing utilisation of virtual consultations to reduce face-to-face follow-up. However, the normal pathway of safeguarding at the point of contact in the ED still applies, but clinicians should remain vigilant with a low threshold of appropriate suspicion as non-verbal and physical clues may be overlooked during virtual consultations. One option is to move from telephonic consultation with the parents to a video-format to examine children, their injuries, and their behaviour visually.

### Limitations and future work

This study, albeit multi-centre in a hotspot of the pandemic, is still not representative of the national picture. Further work is required to look at the national trend. Furthermore, some schools have just started to reopen with the government planning on all children returning to school in September. There needs to be vigilant monitoring to look for signs of a second wave and resurfacing of the pandemic. A future study as we enter a period of gradual relaxation of lockdown measures will provide more data on the continued impact of the pandemic as well as the wider adoption of virtual consultations and active exclusion of delayed presentations of non-accidental injuries.  

## Conclusion

This study represents the early experience of the pandemic at one of the largest central London multi-centre paediatric hospital services. The prevalence of paediatric referrals reduced by nearly two-thirds during the COVID-19 period, with a significant reduction in those being referred for sporting injuries. The COVID-19 pandemic has also changed the pathway for paediatric referrals with greater utilisation of telemedicine in the form of virtual fracture clinics, allowing for safer ongoing follow-up without risking contamination, by reducing face-to-face consultation. These changes may have been triggered by the pandemic due to the necessity of reducing transmission rates, but these may represent novel methods of delivering care to this unique population in the future.

The number of patients requiring operative intervention reduced by two-thirds during the pandemic but an identical 16% of those referred required operative intervention in both 2019 and 2020. Although guidelines at the onset of the pandemic suggested a shift to non-operative treatment of injuries that may previously have required operative intervention, children must be treated based on their clinical needs and the resources available. Treatment must also continue to support and address any concerns the patient or the family may have regarding COVID-19. Neither hospital compromised on providing paediatric patients with evidence-based management for injuries, including surgery within an acceptable timeframe. All clinical needs were met within an acceptable timeframe and there was no need to consider later surgery for possible malunions.

**Table 2.** Risk, prevalence and odds ratios and (95% CI) between post- versus pre-COVID era

Factor	RR or PR (CI)	OR (CI)	p-value **^a^**
Sporting related injury	0.55 (0.36–0.83)	0.43 (0.25–0.74)	0.002
Follow-up	3.1 (1.8–5.3)	3.6 (1.9–6.7)	< 0.001
Clinic pathway			< 0.001
Face to face consultation	0.55 (0.41–0.62)	0.05 (0.02–0.08)	
Virtual fracture clinic	59 (3.5–1,000)	65 (3.7–1,127)	
Discharge from virtual fracture clinic	164 (10–2,664)	224 (14–3,723)	

**^a^**Fisher’s exact test
